# Supplementary material for: Altered microRNA Transcriptome in Cultured Human Liver Cells upon Infection with Ebola Virus
Source: Int J Mol Sci. 2021 Apr 6;22(7):3792. doi: 10.3390/ijms22073792 (PMC8038836; doi:10.3390/ijms22073792)
Supplement: Supplementary file 1 [file ijms-22-03792-s001.zip › Supplementary_File/C_ GO_Analysis_Results/16-30nt_go_Mayinga-96h-Huh7_vs_Control-96h-Huh7_down.mature_mirna_targets/MF_Pvalue_tree.pdf]

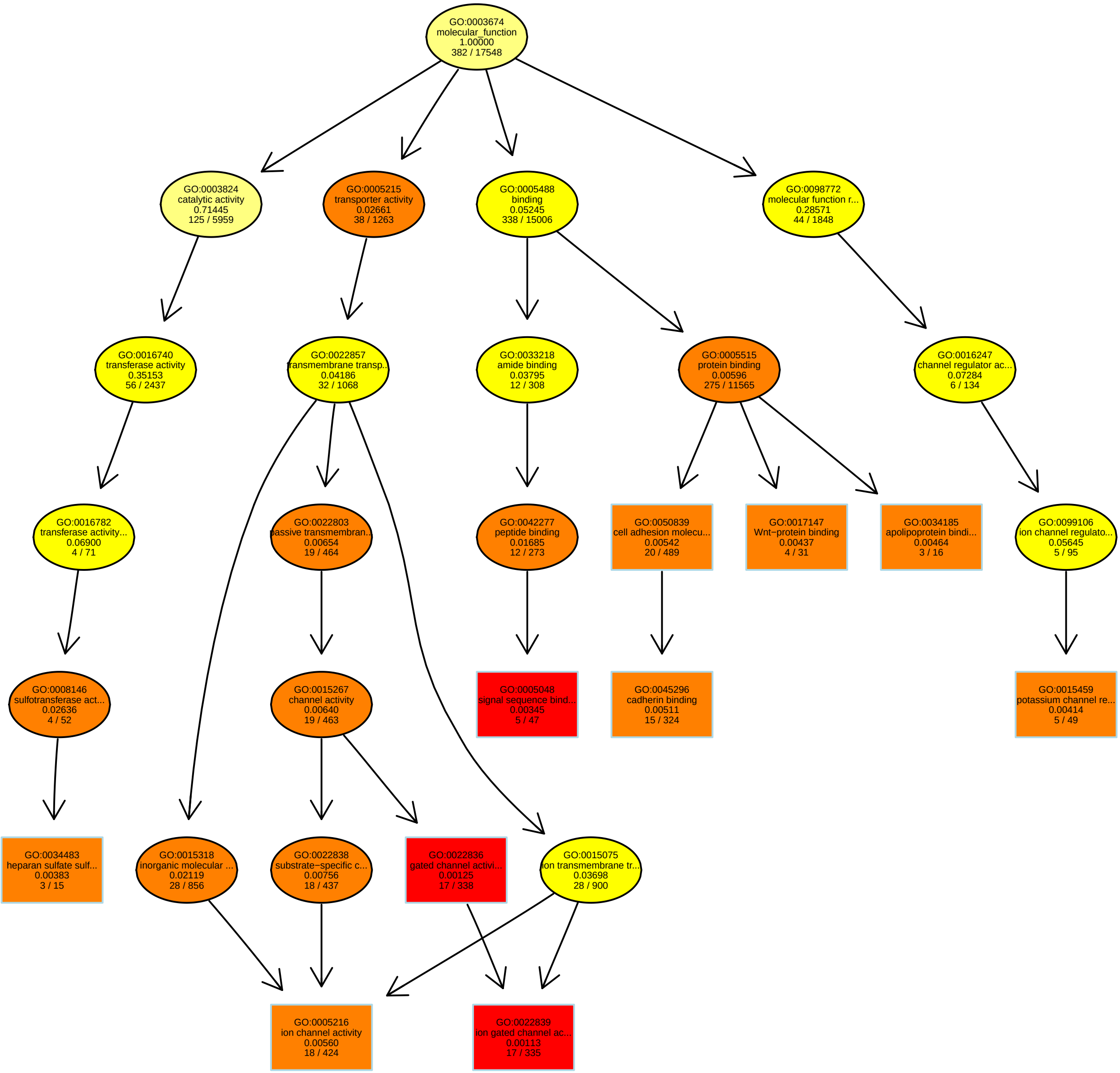

GO:0003674
molecular_function
1.00000
382 / 17548
GO:0003824
catalytic activity
0.71445
125 / 5959
GO:0005215
transporter activity
0.02661
38 / 1263
GO:0005488
binding
0.05245
338 / 15006
GO:0098772
molecular function r...
0.28571
44 / 1848
GO:0016740
transferase activity
0.35153
56 / 2437
GO:0022857
transmembrane transp...
0.04186
32 / 1068
GO:0033218
amide binding
0.03795
12 / 308
GO:0005515
protein binding
0.00596
275 / 11565
GO:0016247
channel regulator ac...
0.07284
6 / 134
GO:0016782
transferase activity...
0.06900
4 / 71
GO:0022803
passive transmembran...
0.00654
19 / 464
GO:0042277
peptide binding
0.01685
12 / 273
GO:0050839
cell adhesion molecu...
0.00542
20 / 489
GO:0017147
Wnt−protein binding
0.00437
4 / 31
GO:0034185
apolipoprotein bindi...
0.00464
3 / 16
GO:0099106
ion channel regulato...
0.05645
5 / 95
GO:0008146
sulfotransferase act...
0.02636
4 / 52
GO:0015267
channel activity
0.00640
19 / 463
GO:0005048
signal sequence bind...
0.00345
5 / 47
GO:0045296
cadherin binding
0.00511
15 / 324
GO:0015459
potassium channel re...
0.00414
5 / 49
GO:0034483
heparan sulfate sulf...
0.00383
3 / 15
GO:0015318
inorganic molecular ...
0.02119
28 / 856
GO:0022838
substrate−specific c...
0.00756
18 / 437
GO:0022836
gated channel activi...
0.00125
17 / 338
GO:0015075
ion transmembrane tr...
0.03698
28 / 900
GO:0005216
ion channel activity
0.00560
18 / 424
GO:0022839
ion gated channel ac...
0.00113
17 / 335
